# Improving access to family planning for women with disabilities in Kaduna city, Nigeria: study protocol for a pragmatic cluster-randomized controlled trial with integrated process evaluation

**DOI:** 10.1186/s13063-023-07892-y

**Published:** 2024-01-05

**Authors:** Sarah Marks, Ekundayo Arogundade, Mark T. Carew, Shanquan Chen, Lena Morgon Banks, Hannah Kuper, Femi Adegoke, Calum Davey

**Affiliations:** 1 International Centre for Evidence in Disability, London School of Hygiene & Tropical Medicine, London, UK; 2 Oxford Policy Management, Abuja, Nigeria; 3 Centre for Evaluation, London School of Hygiene & Tropical Medicine, London, UK

**Keywords:** Family planning, Sexual and reproductive health and rights, Disability, Randomized controlled trial

## Abstract

**Background:**

Globally, women with disabilities are less likely to have access to family planning services compared to their peers without disabilities. However, evidence of effective interventions for promoting their sexual and reproductive health and rights remains limited, particularly in low- and middle-income settings. To help address disparities, an inclusive sexual and reproductive health project was developed to increase access to modern contraceptive methods and reduce unmet need for family planning for women of reproductive age with disabilities in Kaduna city, Nigeria. The project uses demand-side, supply-side and contextual interventions, with an adaptive management approach. This protocol presents a study to evaluate the project’s impact.

**Methods:**

A pragmatic cluster-randomized controlled trial design with surveys at baseline and endline will be used to evaluate interventions delivered for at least 1 year at health facility and community levels in comparison to ‘standard’ state provision of family planning services, in the context of state-wide and national broadcast media and advocacy. Randomization will be conducted based on the health facility catchment area, with 19 clusters in the intervention arm and 18 in the control arm. The primary outcome measure will be access to family planning. It was calculated that at least 950 women aged 18 to 49 years with disabilities (475 in each arm) will be recruited to detect a 50% increase in access compared to the control arm. For each woman with disabilities enrolled, a neighbouring woman without disabilities in the same cluster and age group will be recruited to assess whether the intervention has a specific effect amongst women with disabilities. The trial will be complemented by an integrated process evaluation. Ethical approval for the study has been given by the National Health Research Ethics Committee of Nigeria and London School of Hygiene & Tropical Medicine.

**Discussion:**

Defining access to services is complex, as it is not a single variable that can be measured directly and need for family planning is subjectively defined. Consequently, we have conceptualized ‘access to family planning’ based on a composite of beliefs about using services if needed.

**Trial registration:**

ISRCTN registry ISRCTN12671153. Retrospectively registered on 17/04/2023.

**Supplementary Information:**

The online version contains supplementary material available at 10.1186/s13063-023-07892-y.

## Administrative information

Note: the numbers in curly brackets in this protocol refer to SPIRIT checklist item numbers. The order of the items has been modified to group similar items (see http://www.equator-network.org/reporting-guidelines/spirit-2013-statement-defining-standard-protocol-items-for-clinical-trials/).

**Table:** 

Title {1}	Improving access to family planning for women with disabilities in Kaduna, Nigeria: study protocol for a pragmatic cluster-randomized controlled trial with integrated process evaluation.
Trial registration {2a and 2b}.	ISRCTN registry, ISRCTN12671153. Retrospectively registered on 17/04/2023, https://www.isrctn.com/ISRCTN12671153.
Protocol version {3}	Version 3 (1st November 2022)
Funding {4}	The study is funded by the United Kingdom Foreign, Commonwealth and Development Office under the Programme for Evidence to Inform Disability Action (PENDA) (IATI Identifier: GB-EDU-133903-PENDA). The intervention is designed and implemented under a separate project implemented by a consortium of organizations led by international non-governmental organization (iNGO) Sightsavers and funded by the United Kingdom Foreign, Commonwealth and Development Office under the Disability Inclusive Development programme, as part of Inclusive Futures.
Author details {5a}	Sarah Marks[1]* ^✉^ & Ekundayo Arogundade[2]* (joint first), Mark T. Carew[1], Shanquan Chen[1], Lena Morgon Banks[1], Hannah Kuper[1], Femi Adegoke[2]* (joint last) & Calum Davey[1, 3]* * These authors contributed equally to this work ^✉^Corresponding author [1] International Centre for Evidence in Disability, London School of Hygiene & Tropical Medicine, London, United Kingdom [2] Oxford Policy Management, Abuja, Nigeria [3] Centre for Evaluation, London School of Hygiene & Tropical Medicine, London, United Kingdom
Name and contact information for the trial sponsor {5b}	Not applicable. This is an observational evaluation study that aims to evaluate to what extent the intervention has achieved its goals. The researchers are not involved in the design, allocation or implementation of the intervention—this is being led by Sightsavers.
Role of sponsor {5c}	Not applicable. See above {5b}.

## Introduction

### Background and rationale {6a}

Target 3.7 of the Sustainable Development Goals (SDGs) calls on countries to ‘ensure *universal* access to sexual and reproductive healthcare services, including for family planning, information and education, and the integration of reproductive health into national strategies and programmes’ by 2030 [1]. Access to these services is variable within, as well as between countries, and certain groups are particularly left behind, such as women with disabilities. The United Nations Convention on the Rights of Persons with Disabilities describes people with disabilities as ‘including those who have long term physical, mental, intellectual or sensory impairments which in interaction with various barriers may hinder their full and effective participation in society on an equal basis with others’. Globally, women with disabilities make up approximately 15% of the population and they are less likely to have access to family planning services than other women of the same age [2]. This inequity occurs because women with disabilities experience multiple barriers to accessing family planning services. These barriers broadly include the following: lack of education on sexual and reproductive health; negative family, community and provider attitudes; lack of alternative communication formats; lack of accessible transport and infrastructure; and financial constraints [3, 4]. Consequently, this SDG target will not be achieved unless family planning services are accessible to women with disabilities [5]. Despite this inequity, research on the sexual health of people with disabilities and evidence of effective interventions for promoting their sexual and reproductive health and rights (SRHR) remains limited, particularly in low- and middle-income country (LMICs) settings [6, 7].

Nigeria has 54 million women of reproductive age (15–49 years) [8]. Amongst women in this age group, 0.6% were reported in the 2018 Demographic Health Survey (DHS) to have a disability (Marks, unpublished observations). Moreover, this estimated prevalence does not include any type of psychosocial disability, such as depression or anxiety. Evidence from Lagos State, Nigeria, showed that amongst survey respondents aged 18–75 years old the weighted prevalence of depression and generalized anxiety was 5.5% and 3.5% respectively, with 1.2% experiencing both [9]. The prevalence of disability amongst women of reproductive age in Nigeria is therefore likely to be much higher than 0.6% when psychosocial disabilities are included.

Access to family planning in Nigeria varies by state, with the lowest service coverage in the northern states [10]. DHS data from Kaduna, a northern state with over 9 million people, has shown that just 13.7% of women report currently using a modern method of family planning [10], despite modern contraceptives being generally seen as acceptable across Nigeria [11]. Low uptake in Kaduna has been reportedly due to lack of empowerment amongst women to make decisions on contraception; requirement for husband’s permission to access services; provider insistence on spousal consent; promotion of traditional methods by religious leaders; and high out of pocket expenses—both real and perceived [12]. Utilization of family planning services in Nigeria has also more recently been disrupted by the COVID-19 pandemic, affecting both the demand and supply side [13, 14].

Further analysis of the 2018 DHS data shows that the met need for family planning was almost 10 percentage points higher amongst women without disabilities (28%) than for women with disabilities (19%) (Davey, unpublished observations). Identified barriers to accessing reproductive health services amongst women with disabilities in Nigeria, include significantly lower awareness of family planning [15], as well as impairment-specific barriers. For instance, women with hearing impairment reported embarrassment about asking questions in the presence of an interpreter, and communication difficulties and cost as key barriers to access [16, 17]. Improving access to healthcare requires a holistic approach to address the many dimensions of access [18]. Therefore, correcting this inequality in access and reaching the SDG target in Kaduna will require a comprehensive intervention that is inclusive of women with disabilities.

To help address these disparities, an inclusive sexual and reproductive health project will be designed and delivered by a consortium led by the international non-governmental organization (iNGO) Sightsavers, supported by BBC Media Action under the Disability Inclusive Development (DID) programme, and in partnership with the Joint National Association of Persons with Disability (JONAPWD) and the Network of Disabled Women (NDW) in Nigeria. The Inclusive Family Planning (IFPLAN) project aims to increase access to modern contraceptive methods and reduce unmet need for family planning for women with disabilities aged 15–49 years in Kaduna city, Nigeria. The project’s objectives include increasing knowledge of, intention to use, and perceived support amongst women with disabilities in accessing available family planning services; delivery of inclusive, accessible healthcare services and information by service providers; and meaningful engagement of organizations of persons with disabilities (OPDs) in policy and decision-making processes, and advocacy for disability inclusion in the health sector.

A pragmatic cluster-randomized controlled trial with integrated process evaluation will be undertaken to understand whether the intervention package implemented under the IFPLAN project has increased access to modern contraceptive methods, and reduced unmet need for family planning for women with disabilities. The evaluation, which is being conducted under a separate UK Foreign, Commonwealth, and Development Office (FCDO) programme to the project, is led by London School of Hygiene and Tropical Medicine (LSHTM), in partnership with Oxford Policy Management (OPM) Nigeria under the UK FCDO Programme for Evidence to Inform Disability Action (PENDA).

### Objectives {7}

Informed by the population, intervention, control and outcome (PICO) defined in Table 1, the cluster randomized controlled trial aims to answer the following research questions:

1. Did the intervention increase access to family planning amongst women with disabilities in selected sites in Kaduna city?
2. Did the intervention increase use of family-planning services amongst women with disabilities in selected sites in Kaduna city?
3. Did the intervention reduce the unmet need for family-planning services amongst women with disabilities in selected sites in Kaduna city?

**Table 1:** PICO for cluster-randomized controlled trial of the IFPLAN project

Population	Women aged 18^a^–49 years that report at least ‘a lot’ of difficulty on one of the six questions in the Washington Group short set or ‘daily’ and ‘a lot’ to additional questions on depression or anxiety; women in the same neighbourhood and age range without disabilities, in order to determine whether the intervention has a specific effect amongst women with disabilities
Intervention	Components of the IFPLAN project delivered at the health facility and community levels, in the context of state-wide and national broadcast media and advocacy
Control	‘Standard’ provision of family planning services in the state, in the context of state-wide and national broadcast media and advocacy
Outcome	Primary: Access to family planning Secondary: Knowledge, unmet need, and use of family planning

^a^The evaluation will include women aged 18–49 years, as opposed to the target age of 15–49 years in the IFPLAN project, as it was considered not acceptable to interview women under 18 years regarding their sexual and family planning practices, given the sensitive nature of the research topic and the need for caregiver consent for minors to participate

### Trial design {8}

Evaluation of the IFPLAN project will adopt a pragmatic cluster-randomized controlled superiority trial design with two parallel groups [19]. This is a suitable design since the project funds are limited and there is little evidence available about the efficacy of interventions to improve family planning for women with disabilities in such settings. A cluster design was chosen to reduce the likelihood of contamination between the two arms given that interventions are being delivered at health facility and community levels. A more pragmatic rather than explanatory approach was adopted to the trial in order to meet the needs of decision-makers on the utility of IFPLAN in a real-world implementation setting [20].

The unit of randomization will be the health facility catchment area. Within each cluster women aged 18 to 49 years with disabilities will be recruited by the study team. For each woman with disabilities enrolled, a neighbouring woman without disabilities in the same cluster and age group will be recruited. A baseline survey will be conducted prior to implementation of the intervention in December 2022, with an endline survey after completion of at least 1 year of intervention implementation (anticipated for 2024). The trial will be complemented with an integrated process evaluation to describe intervention implementation as delivered, identify mechanisms of impact and explore context dependencies [21].

## Methods: participants, interventions and outcomes

### Study setting {9}

This trial will be conducted in the region in and around Kaduna city, in Kaduna State.

### Eligibility criteria {10}

Cluster eligibility was based on formative work conducted by Sightsavers to identify functioning health facilities offering family planning services in and around Kaduna city—with a total of 74 facilities identified. All types of operational health facilities that offer family planning services to the general public were eligible, including primary, secondary and tertiary facilities, and public and private facilities. Two of the identified facilities were thus excluded as they were military and air force facilities that did not provide services to the general public.

Facility coordinates were subsequently mapped using QGIS and a 0.5-km radius buffer added to each facility to approximate the catchment area [22]. To reduce the likelihood of contamination between clusters, facilities that were within 0.5 km of each other were merged into the same cluster, so that these health facilities were part of the same arm. The expectation being that if assigned to the intervention arm, intervention activities would be implemented in all health facilities and catchment areas in that cluster. This resulted in 13 clusters with 2–4 facilities (total of 34 health facilities).

**Fig. 1:**
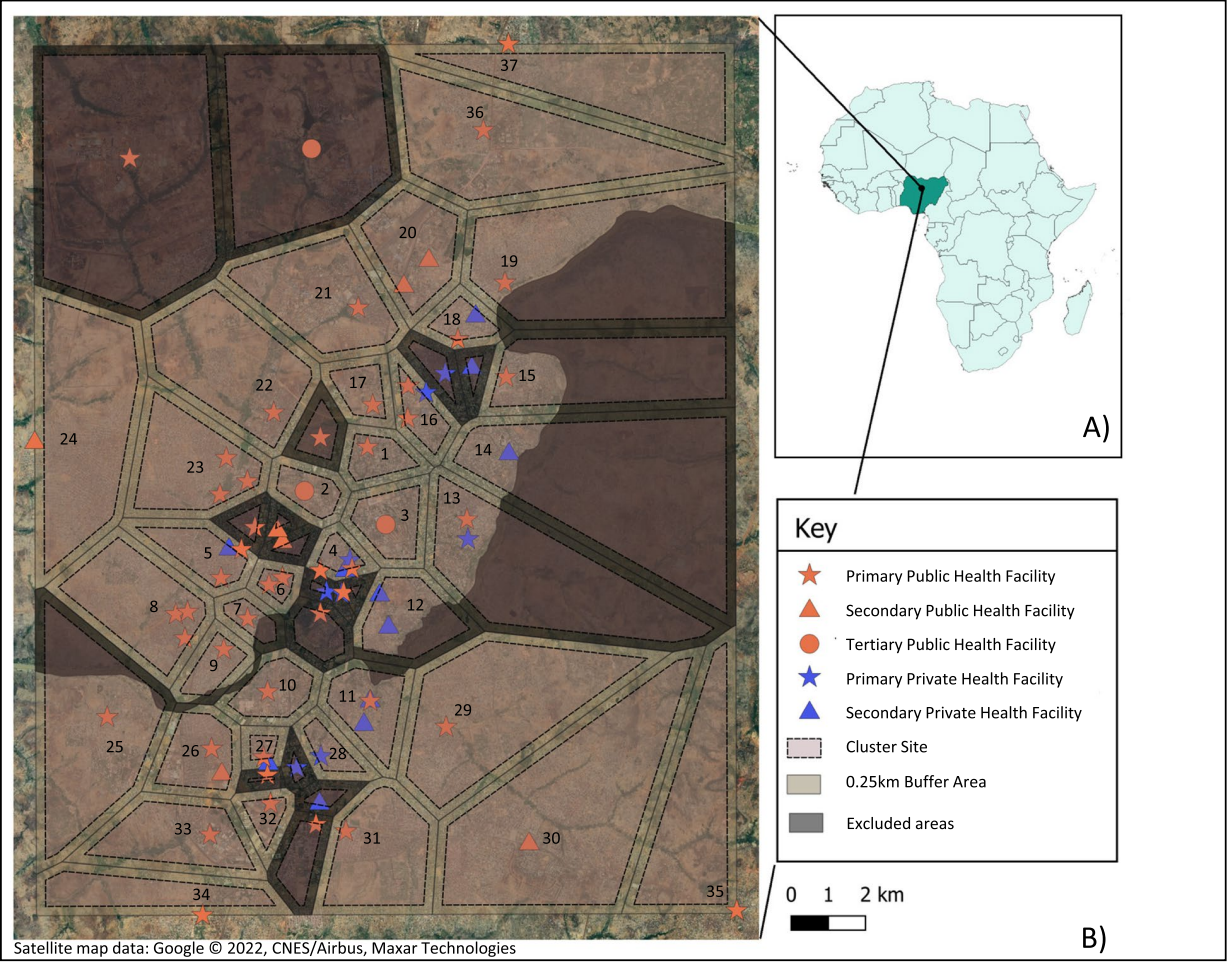
Map of IFPLAN research site in Kaduna City, Nigeria. **A** Map to contextualize the location of Kaduna City within Nigeria and the African continent. **B** Health facilities providing family planning services overlaid onto a satellite map of Kaduna city. The cluster boundaries shown are those generated through the use of a Voronoi overlay in QGIS prior to further refinement based on information from health facilities with regard to the communities in their catchment areas

A Voronoi overlay, whereby polygons are formed around a health facility (or cluster of health facilities) so that the area of the polygon is closest to that health facility (or cluster of health facilities) and no other, was added to the satellite map in QGIS. This was done to get a sense of the geographical areas that might be served by the health facilities in each cluster, and a 0.25 km buffer added between clusters in order to create a combined 0.5 km of separation between clusters. The results of the cluster mapping in QGIS are shown in Fig. 1.

**Fig. 2:**
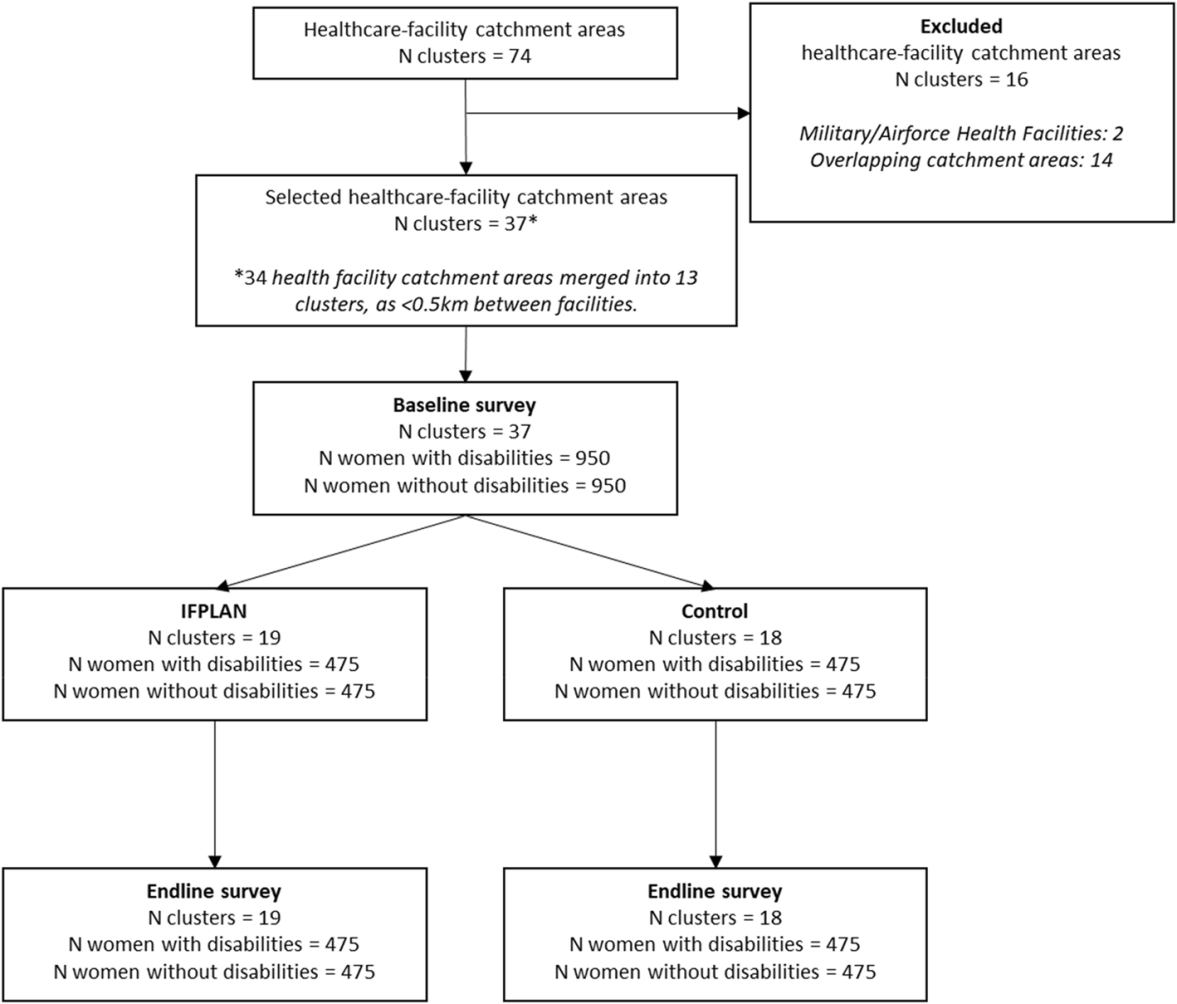
Flow diagram to show the selection of health facility catchment areas

Subsequently, these indicative cluster boundaries were refined by OPM through visits to eligible health facilities. During these site visits, catchment areas were confirmed through discussions with the facility in-charge and verified using the patient register. Communities in catchment areas that either overlapped with health facilities in different clusters or were deemed too insecure by local security experts for the data collection teams to survey were removed. If a health facility’s catchment area completely overlapped with the surrounding facilities, the health facility was removed from the study. The removal of insecure areas will mean that some of the most vulnerable women in Kaduna city will not be included in the trial, which is a limitation of the study. As a result, 13 clusters were excluded from the study, reducing the number of clusters from our desired sample of 38 clusters to 37 eligible clusters in total. A flow diagram to illustrate the selection of health facility catchment areas is outlined in Fig. 2.

The participants in the trial will be women with disabilities, matched with women without disabilities from the same cluster. Participant eligibility will be determined based on the following inclusion criteria: (1) people who identify as women, aged 18–49 years (in this context we do not expect to find any trans men); (2) For women with disabilities, they must report ‘a lot of difficulty’ or ‘cannot do at all’ on any one or more of the six questions in the Washington Group short set or ‘daily’ and ‘a lot’ to additional questions on depression or anxiety from the enhanced question set [23]. The Washington Group questions were chosen as they are a widely used (in at least 75 countries to date), and internationally accepted measure of disability status for population-based surveys [24]; (3) Women without disabilities must be from the same cluster and aged within ± 5 years of the woman with disabilities. Participants will be excluded if they are not able to consent on their own as assessed by the Evaluation to Sign Consent Protocol (see next section)—with the exception of the process evaluation. For the process evaluation, those with severe cognitive or intellectual impairments (or their caregiver) may be included in order to get feedback from people with impairment types that might otherwise be excluded from the trial.

Health facility staff involved in the delivery of family planning services in the study area and intervention implementers will also be interviewed as part of the process evaluation to understand how the intervention was delivered and received.

### Who will take informed consent? {26a}

Informed consent will be sought for all participants before data collection. Potential participants will be provided with information about the research and study procedures by a trained interviewer, recruited by OPM Nigeria. They will have the opportunity to ask questions as part of the informed consent process. For in-person interviews, the information sheet will be either read aloud or given to the participant to read and written consent will be sought (signature or thumbprint if illiterate/blind).

All participants will be above the age of consent. Adults who lack capacity to consent on their own (e.g. people with severe intellectual/cognitive impairments) will be excluded. Capacity to consent for adults will be determined through the ‘Evaluation to Sign Consent’ [25], adapted specifically for the trial and asks four questions to gauge participant’s understanding (see supplementary materials, supplement 1). Participants that are unable to answer the questions even with repeating or re-explaining key parts of the information sheet will be excluded. During the process evaluation, participants that are unable to answer the Evaluation to Sign Consent questions (e.g. due to cognitive or intellectual impairments) may be included to ensure representation of different impairment types. If this is the case, their carer will be asked to complete the consent form, and if possible to still conduct an interview with the person with disabilities directly, they will obtain assent from the individual. Model consent forms can be provided on request.

### Additional consent provisions for collection and use of participant data and biological specimens {26b}

Participants are asked to consent to their anonymized data being shared in a public data repository. There are no consent provisions for biological specimens, as these will not be collected under this trial.

## Interventions

### Explanation for the choice of comparators {6b}

The comparator for the trial will be women in healthcare facility catchment areas receiving standard provision of family planning services. This was deemed a suitable choice for comparison with the intervention arm as it will enable the evaluation to determine if the intervention components of the IFPLAN project have any additional impact on access to family planning amongst women with disabilities compared to currently provided family planning services within the state. Women without disabilities are also included in order to determine whether the intervention has a specific effect amongst women with disabilities or a general effect for those with and without disabilities.

### Intervention description {11a}

The IFPLAN project aims to address multiple barriers to inclusion in family-planning services, using adaptive management and data to target and adjust programme components. The project comprises a range of national, state, and community and facility-based interventions of which this trial will only evaluate the community and facility-based interventions. However, this section describes the whole of IFPLAN so that the community and facility-based interventions can be understood within the entirety of the project.

**Fig. 3:**
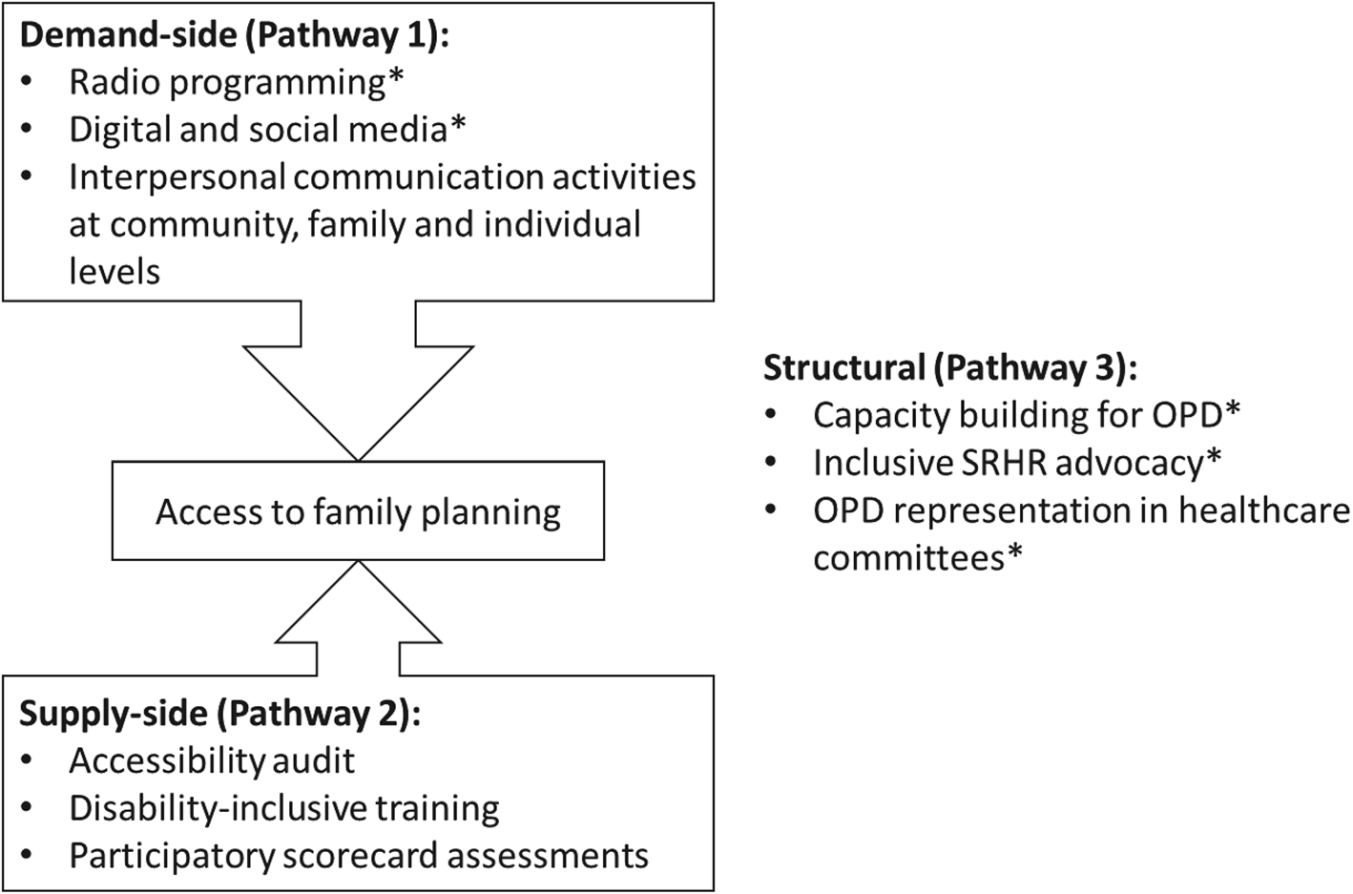
Simplified conceptual framework for the programme. *State and regional level activities (not evaluated by the trial)

The IFPLAN project consortium has developed a full theory of change for how the intervention package is anticipated to work (see supplementary materials, supplement 2), a simplified conceptual framework for which is shown in Fig. 3. This conceptual framework emphasizes how demand- and supply-side components of the project work together to increase access to modern contraception, in the context of efforts to change the structural conditions in which such decisions are made.

The theory of change has three pathways. These are, broadly:

1. Supporting the demand-side by increasing awareness, motivation, support from families/communities, and confidence to access family planning amongst women with disabilities;
2. Supporting the supply-side by changing the service-provision to be more inclusive of persons with disabilities; and
3. Supporting structural change by increasing engagement of OPDs in various aspects of the policy structure.

For each of the three pathways of the theory of change, as summarized in Fig. 3, a range of activities will be conducted to uphold people with disabilities’ rights to have control over their own bodies, fertility and sexuality. Pathway 1 is focused on demand-side changes, informed by a social and behaviour change (SBC) strategy to support people with disabilities to feel able, confident, motivated and supported if and when they choose to use modern contraceptives and practice family planning/child spacing. The SBC strategy has been developed based on existing literature and evidence in Nigeria [10, 12, 26–28], as well as formative research to determine current attitudes and behaviours in Kaduna city and using participatory approaches to define target audiences, and prioritize activities, messages and communication objectives.

The SBC activities include a weekly Hausa language radio drama, complemented by accessible digital and social media content, as well as interpersonal communication activities at the community level. These community-level activities will build on the characters and stories of the radio show, and digital and social media content, and include structured peer-to-peer sessions for people with disabilities to increase knowledge on SRHR and confidence in overcoming barriers to use services, if and when they need them, based on their own informed choices and bodily autonomy; household visits to people with disabilities and structured sessions with family members where needed; structured sessions with *Majalisa*s, existing community assemblies of male heads of households in Northern Nigeria to gain their engagement and support; town hall meetings to gain engagement and support from local leaders; and community plays to educate on SRHR and build on the stories from the radio drama.

The community-level activities will be led by inclusive community-based champions who are trained by Sightsavers and supported by local OPDs. Activities in the structured sessions and town hall meetings will be aided by facilitation guides and content will include (a) the use of an audio device for group listening to radio stories and discussion sessions; (b) the use of a board game to engage young people in the messages, myths and benefits of family planning (adapted from MSI Reproductive Choices to be disability inclusive and suitable for the Nigerian context); and (c) printed materials to aid discussions, such as flip charts and information booklets for young girls, women and men. Prior to implementation, pre-testing of all the materials used in these sessions was conducted with people in two different communities in Kaduna representing a range of impairment types, and focused on the acceptability of the materials.

For supply-side changes under pathway 2, these will be targeted predominantly at primary public health facilities offering family planning services (although secondary and tertiary health facilities, as well as private providers will be included), and focus on three key areas: (1) accessibility audits of and improvements to family planning facilities; (2) disability inclusive training of service providers based on training needs assessments for each health worker cadre, with training conducted by representatives from local OPDs; and (3) participatory scorecard assessments developed by people with disabilities, service providers and decision-makers based on Sightsavers’ Disability Inclusive Scorecard (DISC) tool [29]. DISC is a rights-based tool, aligned with the United Nations Convention on the Rights of Persons with Disabilities (UNCRPD), and brings together people with disabilities, service providers, and local administration to develop and assess indicators to determine the quality of services provided to people with disabilities, collaboratively identifying key gaps and actions required to improve access to family planning for people with disabilities at facility level.

Finally, for structural changes under pathway 3, activities will be focused around capacity building of OPDs, advocacy for inclusive SRHR, and representation of OPDs in healthcare committees.

Interventions under pathway 1 and pathway 2 should increase demand and supply, respectively, and as such increase access to family planning. Pathway 3 is expected to facilitate an increase in access by changing the structures in which demand and supply are met. Pathways 1 and 2 will include activities targeted at the health facilities and health facility catchment areas in the intervention clusters, whereas the activities under pathway 3, as well as some of the activities under pathway 1 (radio, digital, and social media content) will have a broader geographical focus, operating at the state or regional levels. Consequently, this cluster randomized controlled trial will only focus on community and facility-based activities under pathways 1 and 2. Although state- and regional-level activities will be part of the implementation of the IFPLAN project, these will not be part of the evaluation as there will be no means of preventing contamination of the control clusters.

### Criteria for discontinuing or modifying allocated interventions {11b}

Funds may not permit project implementation in all health facilities within the intervention cluster.

### Strategies to improve adherence to interventions {11c}

Intervention implementation will be monitored and an adaptive management approach adopted by the implementing partners to ensure intervention delivery is contextually appropriate and achieves the desired goals of the project [30].

### Relevant concomitant care permitted or prohibited during the trial {11d}

No restrictions on concomitant care.

### Provisions for post-trial care {30}

No specific provisions for post-trial care.

### Outcomes {12}

The primary outcome for the trial, as shown in Table 2, is access to modern family-planning methods. We will also measure ‘realized access’, or *use*, as an important secondary outcome, defined as women aged 18–49 with disability who are sexually active and would like to delay or reduce future pregnancies and are using modern family-planning methods to achieve this.

**Table 2:** Summary of outcomes for the IFPLAN project’s cluster randomized controlled trial

	**Outcome name**	**Measure**
**Primary outcome**	Access to family planning	Survey: binary (yes / no), based on composite of questions: • Know of at least three modern contraceptive methods that are available; & • Know where to access modern family-planning; & name the location; & list the available family planning services; & • Believe that they could use services if they needed them
**Secondary outcome**	Knowledge of family planning	Survey: binary (yes / no), based on composite of questions: • Know of at least three modern contraceptive methods that are available
	Intention to use	Survey: binary (yes / no), based on: • Will use contraceptive method in the future
	Use of family planning (modern method)	Survey: binary (yes / no), based on composite of questions: • Participant or participant’s partner currently doing something or using any method to delay or avoid getting pregnant; & • (Using a modern contraceptive method)
	Unmet need for family planning	Survey: binary (yes / no), based on composite of questions: • Would prefer to delay or not to have another child & • Not using contraception
	Perceived attitudes towards women with disabilities	• Score based on inclusion and participation module of survey

### Participant timeline {13}

Table 3 outlines the time schedule of enrolment, intervention implementation, and assessments for study participants.

**Table 3:** Participant timeline for the IFPLAN project’s cluster randomized controlled trial

Intervention implementation (Planned start April 2023 with implementation lasting for at least 1 year)			
**Visit**	**Visit 1: Baseline survey**	**Visit 2: Process evaluation**	**Visit 3: Endline survey**
**Visit time point**	Prior to intervention implementation	During/post intervention implementation	After intervention implementation
**Visit conducted by**	OPM Nigeria Data collection team	OPM Nigeria Data collection team	OPM Nigeria Data collection team
**ENROLMENT:**			
Eligibility screen	X	X	X
Informed consent of respondent	X	X	X
Resident, Household listing of women 18–49 years	X		X
Women 18–49 years, Washington Group Questions	X		X
**ASSESSMENTS:**			
Women 18–49 years, Respondent’s Background	X		X
Women 18–49 years, Husband’s background	X		X
Women 18–49 years, Marriage and sexual activity	X		X
Women 18–49 years, Fertility preferences	X		X
Women 18–49 years, Reproductive history	X		X
Women 18–49 years, Knowledge of contraceptive methods	X		X
Women 18–49 years, Contraceptive use	X		X
Women 18–49 years with disabilities, Inclusion and participation	X		X
Women 18–49 years with disabilities, Attitudes	X		X
Women 18–49 years, HIV knowledge	X		X
Women 18–49 years, Experience of gender-based violence	X		X
Programme monitoring data on key intervention components		X	
Women 18–49 years, Participant response to family planning services		X	
Women 18–49 years, Unintended consequences of family planning services		X	
Women 18–49 years, Contextual factors affecting use of family planning services		X	
Health facility staff, intervention set-up and delivery		X	
Health facility staff, perceived impact and operation of the intervention		X	
Intervention implementers, intervention set-up and delivery		X	
Intervention implementers, perceived impact and operation of the intervention		X	

### Sample size {14}

Calculations were conducted in Microsoft Excel [31], based on Hayes & Bennett [32]. The assumptions, as summarized in Table 4, were used in determining the likely number of women 18–49 years with disabilities who will be living in the catchment areas of the healthcare facilities, as this is the target population for the evaluation. Various plausible estimates of the between-cluster coefficient of variation (*k*) and effect sizes were explored. Determination of the sample size was guided by the number of areas that the Sightsavers team has capacity and funding to reach, and security concerns limiting implementation to Kaduna city. Assuming *k* = 0.3, a 5% significance level, and 80% power, the trial will be able to detect a risk ratio of 1.5 (i.e. a 50% increase in access compared to the control arm) with 25 women with disabilities interviewed in each cluster. This design will require approximately 20,000 households to be screened for eligible women. It was originally intended to have 19 clusters per arm (a total of 38 clusters overall); however, it was only possible to identify a total of 37 clusters in Kaduna city, consequently 19 clusters were assigned to the intervention arm and 18 clusters to the control arm.

**Table 4:** Assumed and derived values for the IFPLAN cluster randomized controlled trial sample size calculations

**Assumptions:**	
Proportion of women aged 18–49 years	20.0%
Proportion of women 18–49 years who are disabled based on Washington Group Questions^a^	0.7%
Proportion of women 18–49 years with depression or anxiety^b^	6.0%
Estimated overlap between the above	30%
Number of people in each cluster	10,000
Proportion of women who have ‘access’ to family planning	25%
**Derived values:**	
Number of women aged 18–49 years (10,000 × 20%)	2000
Proportion of women 18–49 years who are disabled overall (6% + (0.7% × (100–30%)))	6.49%
Number of women 18–49 years who are disabled (2000 × 6.49%)	130

^a^This is women who report ‘a lot of difficulty’ or ‘cannot do at all’ to one of the six domains in the Washington Group Short Set of questions [23], with this prevalence found in the DHS 2018 data for women 18 to 49 years old in Nigeria

^b^This is the proportion of women who report depression or anxiety using the questions from the Washington Group Enhanced set of questions [23]. This value is based on research in Lagos, in the south of Nigeria [9]

### Recruitment {15}

Health facilities that met the eligibility criteria were recruited to participate in the trial by Sightsavers. For recruitment of the women of reproductive age, female enumerators working for OPM Nigeria conducted a house-to-house survey to find women in the included age range of 18–49 years. Those identified as having a disability based on the Washington Group questions are asked to enroll in the study [23]. The participants are given the opportunity to opt to complete the full survey at a different, and more convenient, time. Loss of time by participants will be the main burden of their involvement in the evaluation. Due to the length of the baseline and endline surveys a non-monetary incentive, such as soap or detergent, will be given to participants.

A neighbourhood-matched sample of women without disabilities will also be interviewed for each woman with disabilities included in the sample; these will be recruited as the next woman of reproductive age within ± 5 years of age, without disabilities, who consents to take part in the study.

## Assignment of interventions: allocation

### Sequence generation {16a}

Cluster allocation was conducted through a random draw. The first 19 clusters drawn were allocated to the intervention arm and the remaining 18 clusters allocated to the control arm.

### Concealment mechanism {16b}

Cluster numbers were hidden during allocation using concealed cards.

### Implementation {16c}

Intervention assignment was conducted at one time point before the start of the trial during a multi-stakeholder event organized by Sightsavers for implementation of the IFPLAN project. The random draw was conducted by government officials in Kaduna during the event.

## Assignment of interventions: Blinding

### Who will be blinded {17a}

No blinding amongst participants, data collectors, or data analyst. Given the nature of the intervention, health facilities in clusters assigned to intervention or control will be aware of their allocation and women in the catchment area may also be aware of the health facility or their community’s allocation—making it impossible to maintain blinding of the data collectors. Given that there are also 19 clusters in the intervention arm and 18 clusters in the control arm, blinding of the data analyst will not be possible.

### Procedure for unblinding if needed {17b}

Not applicable, as there is no blinding in the trial unblinding will not occur.

## Data collection and management

### Plans for assessment and collection of outcomes {18a}

Data will be collected by experienced and trained female enumerators, overseen by OPM Nigeria. All data collection tools and procedures will be pre-tested and piloted prior to use.

The trial will collect baseline and endline data from all of the health facility catchment areas included in the study using household surveys. The baseline survey will be conducted prior to implementation of the project interventions and the endline survey will be conducted after at least 1 year of IFPLAN activity implementation.

Project monitoring data will be routinely collected by implementing partners to support intervention implementation and shared with LSHTM for the purposes of the process evaluation. During and/or after the IFPLAN project, semi-structured interviews will be undertaken with women of reproductive age with disabilities in the intervention arm to understand participant responses to the interventions, identify any unintended consequences, and compare their experiences to women without disabilities in the intervention arm, and women with disabilities in the control arm (approximately 30 interviews conducted in total). Furthermore, key health facility staff and intervention implementers will be interviewed to elucidate mechanisms of impact and contextual factors for implementation.

### Plans to promote participant retention and complete follow-up {18b}

Adaptations will be put in place to support the direct participation of people with different impairments in data collection. For example, sign language interpretation or options to provide written responses will be made available to people with profound hearing impairments. Simplified information sheets will be used for people with cognitive or intellectual impairments in the process evaluation.

### Data management {19}

Data entry for the baseline and endline surveys will be conducted electronically using tablets, with digital forms programmed using SurveyCTO [33], replicating the paper forms approved by the Institutional Review Boards. Validation and consistency checks will be programmed into the data collection forms and applied at the point of data entry into a specific field. Collected data will be password protected on devices and servers.

For the process evaluation, interviews will be audio recorded, alongside written interview notes. Interviews will be recorded, transcribed, and translated into English as soon as possible after the interview. A sample of interview recordings from each interviewer will be checked to ensure that these were conducted in accordance with the topic guide and qualitative interview best practices. Anonymized project monitoring data shared with LSHTM will be reviewed and relevant data extracted by the study team for the purpose of the process evaluation.

### Confidentiality {27}

Field staff will sign a confidentiality agreement to not disclose any information outside of the study, unless for safeguarding purposes. Given that some of the questions in the survey relate to gender-based violence, if any women disclose that they have been a victim of gender-based violence or the enumerator suspects that they might be, the enumerators will confidentially refer the respondent to local organizations that support victims of gender-based violence and discreetly share a small information card with the organizations’ contact information. Paper consent forms with participant names will be stored separately to the survey data and stored in a locked cabinet. Collected data will be anonymized using unique identification numbers.

### Plans for collection, laboratory evaluation, and storage of biological specimens for genetic or molecular analysis in this trial/future use {33}

Not applicable, no biological specimens collected.

## Statistical methods

### Statistical methods for primary and secondary outcomes {20a}

To test the effect of the intervention on women with and without disability, analyses will be initially conducted separately for each group. To determine whether the interventions have any specific effect on women with disability, in comparison to those without disability, data for women with and without disability will be analysed together with an interaction term between intervention status (yes or no) and disability status (yes or no).

The primary outcome will be modeled as access to family planning as defined in Table 2, the proportion for which will be reported for each arm. As the primary and secondary outcomes are based on dichotomous measures with grouping of respondent outcomes within health facility catchment areas, multilevel logistic regression will be used to investigate the association between disability status with the outcomes of interest. Basic respondent and health facility catchment area characteristics will be used in the regression, in addition to random-effects at the cluster level. Any other variables that appear to be imbalanced at baseline will be included in an adjusted analysis that will be reported separately.

### Interim analyses {21b}

Baseline data will be analysed to determine whether key characteristics are evenly distributed between control and intervention arms, and to inform IFPLAN project implementation indicators. We will first assess and report the balance at baseline between the intervention and control arms based on socio-economic factors and any other factors likely to affect the implementation of the intervention.

### Methods for additional analyses (e.g. subgroup analyses) {20b}

Subgroup analysis will be conducted focusing on women identified as having functional difficulty (with or without depression or anxiety). This analysis will be based on the same analytical approach as outlined for the primary and secondary indicators.

For the process evaluation, thematic analysis will be conducted on translated interview transcripts based on project theory, process evaluation domains, and emerging themes on access to family planning. A comparative case study approach will be utilized to compare key themes between women with and without disabilities to better understand the mechanisms of impact for the project and unintended consequences. The comparative case studies and interview data from key health facility staff and intervention implementers will be complemented by programme monitoring data reviewed and synthesized by the study team to further understand the fidelity, dose, adaptions and reach of the interventions implemented.

### Methods in analysis to handle protocol non-adherence and any statistical methods to handle missing data {20c}

We will adopt an intention-to-treat analysis, and therefore health facility catchment areas will be analysed per their original arm assignment even if subsequently the intervention is not implemented in any or all of the health facilities in that cluster. Given that the health facility catchment areas are close together due to the small geographical area of Kaduna city, respondents will be asked about which facility they attend for family planning services and their participation in community-based family-planning activities in order to conduct a sensitivity analysis using an as-treated per-protocol analysis. Furthermore, should health facility staff be transferred between facilities from the intervention arm to control arm (or vice versa) this will be captured through the process evaluation and its potential effects explored.

### Plans to give access to the full protocol, participant-level data and statistical code {31c}

Data will be made available on LSHTM’s Data Compass [34], along with project documentation and a data-users guide 12 months after the end of the study.

## Oversight and monitoring

### Composition of the coordinating centre and trial steering committee {5d}

LSHTM is responsible for the study design of this observational trial. The co-Principal Investigators are Dr Calum Davey and Professor Hannah Kuper at LSHTM, supported by Morgon Banks, Mark Carew, Shanquan Chen and Sarah Marks as co-investigators. Femi Adegoke and Ekundayo Arogundade are co-investigators based at OPM Nigeria, who will be managing data collection.

Design, delivery and allocation of the interventions is led by Sightsavers and supported by a Steering Committee that includes representatives from OPDs and government.

### Composition of the data monitoring committee, its role and reporting structure {21a}

Project monitoring will be led by Sightsavers, who will compile data from implementing partners to monitor intervention delivery.

### Adverse event reporting and harms {22}

Delivery of interventions will be monitored by Sightsavers; however, no severe adverse events are anticipated. The IFPLAN project only seeks to provide awareness of family planning options, how these can be accessed, and improving the accessibility of family planning services in Kaduna city. Interventions have been developed in close partnership with people with disabilities and the project aims for women to meet their own family planning needs, focusing on bodily autonomy and the rights of people with disabilities.

Sightsavers has a mechanism which is open to the public to report improper conduct through the Speak Up platform [35]. If anyone witnesses or suspects any safeguarding issues or misconduct within the project, they can report it anonymously using this platform—and an investigation will follow.

### Frequency and plans for auditing trial conduct {23}

Field supervisors will be responsible for quality assuring that work by the data collection teams is conducted per protocol and the data manager will conduct daily checks for the completeness, accuracy, and timeliness of data, following up with the data collection teams as required. During data collection, daily debrief sessions will be conducted to review work conducted.

### Plans for communicating important protocol amendments to relevant parties (e.g. trial participants, ethical committees) {25}

Modifications to the protocol that affect the conduct of the trial will be agreed upon by LSHTM and OPM Nigeria, and approved by the LSHTM Ethics Committee and the National Health Research Ethics Committee of Nigeria prior to implementation. Important protocol modifications will be updated on the trial registry.

### Dissemination plans {31a}

Findings from the trial will be shared with key stakeholders in Nigeria and published in peer-reviewed journals.

## Discussion

There is currently a dearth of information regarding effective interventions for inclusive access to sexual and reproductive health services for women with disabilities in LMICs [7]. However, defining access to services is complex, as it is not a single variable that can be measured directly [18]. Use of services is not equivalent to access, since (1) not everyone has an objective ‘need’ for services and (2) not everyone who has a need has a desire—or subjective ‘need’—to use services. For family planning, we might define objective need, (1), as fecund women who report that they would like to delay or permanently end having children while also having sexual relations with a fertile man. This raises a secondary issue, which is that this ‘objective’ need is based on the *subjective* desires that the woman has regarding family planning. Unlike for a condition that can be objectively assessed—such as HIV status—the need for family planning has only subjective components, both in determining that there is an issue that could be remedied with use of services, (1), and the desire to use services to address this need (2). Use is therefore realized access [18]. Unlike HIV, there is no comparable ‘treatment cascade’ either, since for any position on a cascade there is a reasonable choice, for example between having more children or not. In the context of persons with disability, this is particularly important when considering the position of women with disability who might be pressured into considering contraceptive methods against their will [36]. A further complication is that a woman’s desires for family-planning services can change over time, and so it is not possible to use her position at baseline as a guide to what ‘should’ be her position at endline.

Access is made up of several supply- and demand-side factors [18], with additional factors that affect only persons with disability [3, 4]. A complete assessment of access could ascertain whether someone who had a need and desire for service would be able to use them. However, this would only be successful if our model was correct, which for persons with disability, and many possible impairments, is unlikely. If the model is not correct then, for example, we might say that someone ‘has access’ to services when, in fact, there remained impediments (or lack of support factors) that would render the services inaccessible. If some of the impediments have been removed, then we could say her access has ‘improved’, while being aware that access has not been realized.

We have conceptualized ‘access to family planning’ for the purposes of this trial as shown in Table 2, to try to address these issues. This conceptualization takes account of the following characteristics of access in the context of this family-planning intervention:

- All women should know about available services if they are to have ‘access’;
- Women who want to delay or reduce future pregnancies should be able to realize ‘access’ through use of services;
- Women who do not want to delay or reduce future pregnancies, or are not in sexually active relationships, are not expected to have realized their ‘access’ to services through service use; instead, understanding access is a composite of their beliefs about using services if they needed them (hypothetically) and their impressions of the barriers or facilitators that we expect should determine use.

There remains a challenge with this conceptualization. Part of the effects of the intervention could be to increase voluntary demand for family planning, leading to an increase in the number of women who would like to delay or reduce future pregnancies. This might occur without a compensatory increase in the availability of services since the intervention has only limited influence over the provision of services. Demand will be included as a secondary outcome in anticipation of this eventuality.

Despite the challenges in measuring access, it is hoped that the evidence generated by this trial strengthens the evidence base on how to improve inclusive family planning for women with disabilities in Nigeria and other LMIC settings.

## Trial status

This manuscript is based on protocol version 3, 01 November 2022. Baseline recruitment and data collection started in December 2022. Endline and process evaluation recruitment and data collection is anticipated for 2024.

### Supplementary Information

**Additional file 1: Supplement 1. **Evaluation to Sign Consent Protocol. **Supplement 2.** Theory of change for the IFPLAN intervention, as devised by the project consortium.

## Data Availability

Data will be made available on LSHTM’s Data Compass 12 months after the end of the study, along with project documentation and a data-users guide [34]. The data will be made available open access, ensuring that no identifiers are included in the data. Explicit consent has been included for making data open access.
