# Supplementary material for: Improving access to family planning for women with disabilities in Kaduna city, Nigeria: study protocol for a pragmatic cluster-randomized controlled trial with integrated process evaluation
Source: Trials. 2024 Jan 5;25:28. doi: 10.1186/s13063-023-07892-y (PMC10768103; doi:10.1186/s13063-023-07892-y)

**Supplementary Materials**

**Supplement 1: Evaluation to Sign Consent Protocol**

Capacity to consent for adults will be determined through the “Evaluation to Sign Consent”[[1]](https://lshtm-my.sharepoint.com/personal/lshsm11_lshtm_ac_uk/Documents/3.%20PENDA%20-%20T049/Supplementary%20Materials_TO49%20Trials%20Protocol%20Manuscript.docx#_ftn1) . This protocol (adapted for this study) asks the following 4 questions to participants to gauge their understanding:

1. What is expected from you if you participate in this study? (Acceptable answer: answer questions about family planning services)

2. What is a potential risk to participating? (Acceptable answer: I might feel discomfort talking about my experiences)

3. What is the benefit to participating? (Acceptable answers: Information I provide can help improve policy and programmes in the future)

4. What if you don’t want to continue? (Acceptable answer: ask to stop)

Participants that are unable to answer the above questions even with repeating/re-explaining key parts of the information sheet will be excluded. During the process evaluation, participants that are unable to answer the above questions may be included and if this is the case, their carer will be asked to complete the consent form If possible to still conduct an interview with the person with disabilities directly, they will obtain assent for the individual.

[[1]](https://lshtm-my.sharepoint.com/personal/lshsm11_lshtm_ac_uk/Documents/3.%20PENDA%20-%20T049/Supplementary%20Materials_TO49%20Trials%20Protocol%20Manuscript.docx#_ftnref1) Resnick, B., et al., Reliability and validity of the evaluation to sign consent measure. The Gerontologist, 2007. 47(1): p. 69-77.

**Supplement 2: Theory of change for the IFPLAN intervention, as devised by the project consortium.**


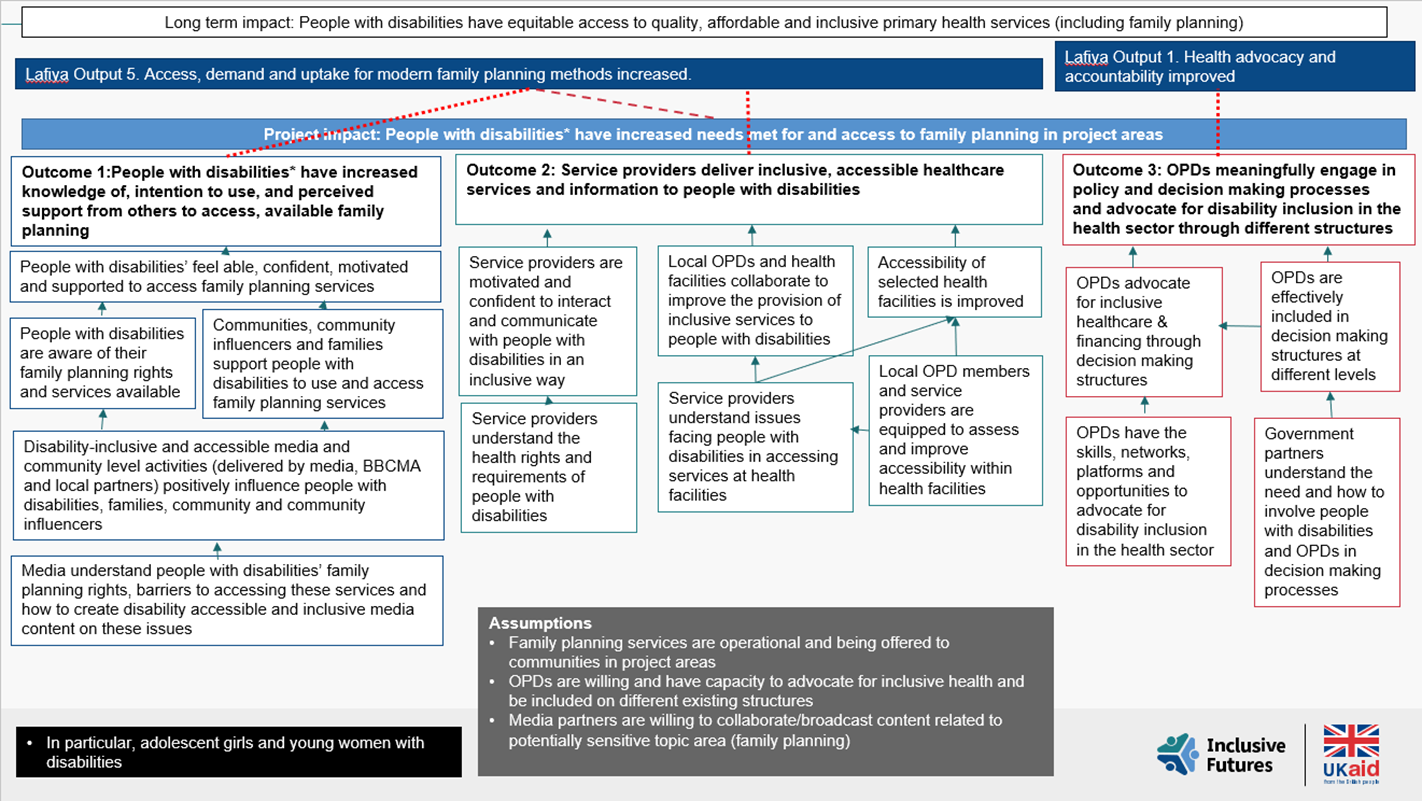

Supplement: Supplementary file 1 — Additional file 1: Supplement 1. Evaluation to Sign Consent Protocol. Supplement 2. Theory of change for the IFPLAN intervention, as devised by the project consortium. [file 13063_2023_7892_MOESM1_ESM.docx]
